# Intravitreal dexamethasone implant Ozurdex® in naïve and refractory patients with different subtypes of diabetic macular edema

**DOI:** 10.1186/s12886-018-1022-9

**Published:** 2019-01-11

**Authors:** Verónica Castro-Navarro, Enrique Cervera-Taulet, Catalina Navarro-Palop, Clara Monferrer-Adsuara, Laura Hernández-Bel, Javier Montero-Hernández

**Affiliations:** 1 University General Hospital of Valencia, Avenida Tres Cruces S/N, 46015 Valencia, Spain; 2 Ophthalmology Department, Avenida Tres Cruces S/N, CP/46015 Valencia, Spain

**Keywords:** Diabetes, Macular edema, Corticoids, Dexamethasone implant, Ozurdex

## Abstract

**Background:**

Diabetic macular edema (DME) can be treated with different alternatives, among them Dexamethasone intravitreal implant 0.7 mg (DEX 0.7) has demonstrated that may improve both central macular thickness (CMT) and best corrected visual acuity (BCVA). This study aimed to evaluate the effect of the intravitreal dexamethasone implant Ozurdex® in patients with different subtypes of diabetic macular edema over a 6-month follow-up period.

**Methods:**

Eighty-four (29 naïve and 55 previously treated) eyes were included in this retrospective study. For each patient, the BCVA [Early Treatment Diabetic Retinopathy Study (ETDRS) charts] and macular thickness on optical coherence tomography (OCT) at baseline visit and within the 2nd, 4th, and 6th months of follow-up were obtained. The main outcomes measurements were the mean change in BCVA and in CMT with respect to the baseline value. The percentage of patients gaining ≥5 letters and ≥ 10 letters in BCVA was also analyzed. Results: A total of 84 eyes, 29 (34.5%) naïve and 55 (65.5%) non-naïve, from 69 patients were included in the study. BCVA at baseline was 58.8 (16.4) and 61.8 (11.6) in naïve and refractory patients, respectively, *p* = 0.4513. At every visit, BCVA significantly improved from baseline in naïve and non-naïve eyes (*p* < 0.0001 and *p* = 0.0003, respectively; Friedman rank sum test). At baseline, the mean CMT was 466.2 (189.7) μm and 448.1 (110.7) μm in the naïve and non-naïve patients, respectively (*p* = 0.5830); and decreased to 339.3 (92.5) μm and 357.5 (79.1) μm, respectively (*p* = 0.0004 and *p* < 0.0001, respectively, Wilcoxon signed-rank test). The proportion of patients gaining ≥10 letters was significantly greater in the naïve group, *p* = 0.0199.

**Conclusion:**

The intravitreal dexamethasone implant (Ozurdex) is effective for the treatment of diabetic macular edema, even in refractory cases that have failed to respond to previous therapies.

## Background

Macular edema can be considered one of the most important issues in retinal pathologies as damage to the macula has an immediate effect on central visual acuity and may substantially affect a patient’s quality of life [1].

Diabetic macular edema (DME), a macular thickening secondary to diabetic retinopathy (DR) that may be present in any of the stages of this disease, results from a blood-retinal barrier defect that leads to vascular leakage, fluid accumulation, and macula thickening [2]. This breakdown is the result of the expression of inflammatory factors [3], including vascular endothelial growth factor (VEGF), intercellular adhesion molecule-1, interleukin-6, monocyte chemotactic protein-1 and leukostasis [4].

According to the optical coherence tomography (OCT) it is possible to define three different morphologic subtypes of macular, namely Sponge-like diffuse retinal thickening (DRT), cystoid macular edema (CME) and serous retinal detachment (SRD) [5, 6].

Besides glycemic control, different treatment alternatives exist for patients presenting with DME, including laser photocoagulation, which has been the mainstay of treatment for patients with DME for the past four decades [7–9].

Over the past decade, advances in elucidating the pathogenesis of DME has led to new therapies, including anti-vascular endothelial growth factor (VEGF) agents and corticosteroids [7, 8]. Intravitreal anti-VEGF agents, either alone or as an adjunct to laser photocoagulation have emerged as a treatment for DME [7, 8]. Although the Food and Drug Administration (FDA) has approved their use for the treatment of DME [10, 11], not all patients respond to the treatment and the compliance to the treatment is not high because of the numerous required injections during the year [12, 13].

Intravitreal corticosteroids block production of inflammatory mediators, such as VEGF, and inhibit leukostasis [14, 15].

Dexamethasone, an anti-inflammatory agent, has the highest relative clinical efficacy of any corticosteroid applied to ophthalmological practice [9].

Dexamethasone intravitreal (DEX) implant (0.7 or 0.35 mg) (Ozurdex, Allergan, Inc., Irvine, CA, USA), consists of micronized dexamethasone in a biodegradable copolymer of polylactic-co-glycolic acid which slowly releases steroids into the vitreous over a period of about 6 months [16, 17]. In 2014, Based on the results of the MEAD study [18], the FDA and most European countries approved Ozurdex for the treatment of DME.

Different studies have demonstrated that Ozurdex® may improve the central macular thickness (CMT) and best corrected visual acuity (BCVA) in DME patients [18–31].

Moreover, in eyes with DME, DEX implants provide meaningful functional benefits as soon as 1 month after treatment [24].

There is not too much evidence addressing the impact of the DME subtype on the treatment outcomes. It was suggested that in patients with DME treated with a single intravitreal bevacizumab injection CME and SRD subtypes were associated with a greater reduction in the CFT than the DRT subtype, although the changes in BCVA did not significantly differ between groups [32]. Additionally, it was reported that the morphologic subtypes with OCT were considered a good prognostic factor of treatment effectiveness in DME [33].

The purpose of this study was to evaluate the effect of the intravitreal dexamethasone implant Ozurdex® on the CMT and BCVA in naïve and refractory patients with diabetic macular edema. Additionally, the effect of the DEX implant 0.7 mg according to the DME subtype was analyzed.

## Methods

A retrospective, comparative, and single center study was conducted on consecutive patients with DME, either naïve or refractory, who received one or more dexamethasone implants, between January 2012 and May 2017, and followed up for at least 6 months.

The study protocol was approved by the ethical research committee of the University General Hospital of Valencia, which waived the need for written informed consent of the participants. The ethical principles outlined in the Declaration of Helsinki and those of Good Clinical Practice were followed.

Inclusion criteria were a diagnosis of type 1 or 2 diabetes and center-involved DME treated with one or more intravitreal DEX implants and followed up for at least 6 months and a BCVA ≥30 and ≤ 80 letters [Early Treatment Diabetic Retinopathy Study (ETDRS) charts]. Patients with ME secondary to a cause other than diabetes mellitus, macular ischemia, a history of vitrectomy, intraocular surgery in the previous 6 months, a history of systemic corticosteroids within 6 months before baseline evaluation, uveitis, glaucoma or ocular hypertension, dense cataract, and those lost to follow-up were excluded from the study. Patients with macular ischemia were excluded for their poor prognosis, which might distort the results.

Fluorescein angiography (FA) and Spectral Domain-optical coherence tomography (SD-OCT) B-scans and volume scans images were obtained with 3D-TopCon (3D OCT-2000 Spectral Domain OCT, Topcon Medical Systems, Inc., Oakland, USA). CMT and macular volume (MV) were automatically generated. Two doctors of the Ophthalmology Department (VCN & CMA), who were unaware of BCVA or other information about the eyes, divided the DME among the different subtypes described by Otani et al. [5] and assessed non-perfusion areas on FA and active exudation.

According to the appearance of DME on SD-OCT images, the patients were divided into three subgroups [6]: a) The DRT type when a sponge-like retinal swelling of the macula with reduced intraretinal reflectivity was present; b) The CME type when in the macular area were present intraretinal minimally reflective round or oval spaces of with highly reflective septa separating cystoid-like cavities; and c) The SRD type when a nonreflective space between the pigment retinal epithelium and the neurosensory retina was observed. If DRT was combined with CME or SRD, the pattern was classified as either CME or SRD, as appropriate, and when DRT, CME, and SRD were all present, the type was classified as SRD.

In the previously treated patients, previous anti-VEGF injections (ranibizumab and aflibercept) were administered on a pro renata (PRN) basis, according to specific retreatment criteria, including loss of visual acuity (VA) of more than one Snellen line, increase in CMT of > 50 μm, and presence of intraretinal (IRF)/subretinal (SRF) fluid compared to the previous visit. Refractory was considered when despite at least three consecutive anti-VEGF (ranibizumab or aflibercept) injections applied once a month with no or partial response (worsening of BCVA by 2 ETDRS lines or reduction of less than 10% of retinal thickness or reduction of central subfield macular thickness less than 50 μm) was observed. When refractory was observed, no switching among anti-VEGF injections was performed.

DEX implant 0.7 mg was injected into the vitreous cavity using standard protocols [23].

For each patient, the BCVA [Early Treatment Diabetic Retinopathy Study (ETDRS) charts] and macular thickness on optical coherence tomography (OCT) at baseline visit and within the 2nd, 4th, and 6th months of follow-up were obtained. After the DEX implant injection, retreatment was judged necessary at the scheduled follow-up visits if CMT > 250 μm in the central subfield and IRF or SRF were present or if BCVA decreased due to recurrence of macular edema.

The main outcomes measurements were the mean change in BCVA and in CMT with respect to the baseline value. Secondary outcome measures included the percentages of patients gaining ≥5 letters and ≥ 10 letters in VA and the percentage of those with edema resolution at the end of the 6-month follow-up.

Group analyses were performed by comparison of treatment naïve versus non-naïve eyes.

### Statistical analysis

A standard statistical analysis was performed using MedCalc Statistical Software version 18.5 (MedCalc Software bvba, Ostend, Belgium; http://www.medcalc.org; 2018).

Before the study, it was determined that a sample of at least 27 patients per group was required to detect a difference greater or equal than 5 letters in ETRDS between naïve and Non-naïve eyes, at a significance level of 0.05, with a power of 0.90; and assuming a standard deviation of 5.5. Descriptive statistics [mean (standard deviation)] and percentages were used, as needed.

Data were tested for normal distribution using a D’Agostino-Pearson test.

If data were normally distributed, a repeated measures ANOVA and the Greenhouse-Geisser correction was used for the determination of the changes in visual acuity and in macular thickness into the groups. We used a linear mixed model in order to consider the correlations between the repeated measures and the existence of missing data. If data were no normally distributed, the comparisons of the changes in visual acuity and in macular thickness were performed using a Friedman’s two-way analysis test. The Wilcoxon signed-rank test was used for the determination of the changes in visual acuity and in macular thickness into the groups; while the Mann–Whitney U test was used in the evaluation of the changes in visual acuity, the changes in macular thickness, and time to recurrence between naïve and non-naïve eyes. The Kruskal-Wallis test was used to evaluate the impact of DME subtype on the functional and structural results.

The Chi-squared test was used in the evaluation of the distribution of the patients with recurrence and in the patients gaining ≥5 letters and ≥ 10 letters in BCVA.

## Results

A total of 84 eyes, 29 (34.5%) naïve and 55 (65.5%) non-naïve, from 69 patients were included in the study. According to the appearance of DME on SD-OCT images, the study included 20 (23.8%) eyes with DRT; 41 (48.8%) eyes with CME and 23 (27.4%) eyes with SRD. Principal baseline clinical and demographic characteristics are summarized in Table 1.

**Table 1:** Baseline clinical and demographic characteristics in the overall study population

Characteristic	Total (*n* = 84)	Naïve (*n* = 29)	Nonnaïve (*n* = 55)	P^a^
Age, years				
Mean (SD)	71.1 (9.2)	73.6 (9.1)	69.7 (9.1)	0.0465
95% CI	69.1 to 73.1	70.3 to 77.2	69.7 to 76.4	
HbA1c, (%)				
Mean (SD)	7.6 (0.9)	7.4 (0.9)	7.6 (1.0)	0.2575
95% CI	7.4 to 7.8	7.1 to 7.8	7.4 to 7.9	
Sex, n (%)				
Man	58 (69.0)	16 (55.2)	42 (76.4)	0.0471^b^
Woman	26 (31.0)	13 (44.8)	13 (23.6)	
Eye, n (%)				
Right	45 (53.6)	17 (58.6)	28 (50.9)	0.5030^b^
Left	39 (46.4)	12 (41.4)	27 (49.1)	
Type of DME, n (%)				
DRT	20 (23.8)	6 (21.4)	14 (25.5)	
CME	41 (48.8)	12 (42.9)	28 (50.9)	0.5089^b^
SRD	23 (27.4)	10 (35.7)	13 (23.6)	
Visual Acuity*				
Mean (SD)	60.8 (14.6)	58.8 (16.4)	61.8 (13.6)	0.4513
95% CI	57.6 to 63.9	52.6 to 65.0	58.2 to 65.5	
CMT, μm				
Mean (SD)	448.1 (110.7)	466.2 (189.7)	448.1 (110.7)	0.7348
95% CI	418.2 to 478.0	394.0 to 538.3	418.2 to 478.3	
Number of anti-VEGF injections				
Mean (SD)	N.A.	N.A.	7.0 (3.9)	N.A.
95% CI			5.9 to 8.0	

*SD* standard deviation, *CI* confidence interval, *DME* diabetic macular edema, *DRT* diffuse retinal thickness, *CME* cystoid macular edema, *SRD* Serous retinal detachment, *CMT* central macular thickness, *VEGF* vascular endothelial grow factor, *NA* Not applicable

^a^Mann-Whitney U test (between naïve and non-naïve patients)

^b^Chi-square test

^*^Letters in the Early Treatment Diabetic Retinopathy Study (ETDRS) charts

According to the DME subtype, with the exception of the CMT, that was significantly lower in DRT patients (*p* < 0.0001), there were no significant differences in the baseline demographic or clinical characteristics (Table 2).

**Table 2:** Main baseline demographic and clinical characteristics according to the diabetic macular edema (DME) subtypes

Characteristic	DRT (*n* = 20)	CME (*n* = 41)	SRD (*n* = 23)	P^a^
Age, years				
Mean (SD)	72.5 (7.2)	72.4 (8.7)	67.6 (10.9)	0.0987
95% CI	69,1 to 75,8	69.6 to 75.2	62.9 to 72.3	
HbA1c, (%)				
Mean (SD)	7.4 (0.9)	7.6 (1.0)	7.7 (0.8)	0.7371
95% CI	7.0 to 7.9	7.3 to 7.9	7.3 to 8.0	
Sex, n (%)				
Man	14 (70.0)	25 (61.0)	19 (82.6)	0.1981^b^
Woman	6 (30.0)	16 (39.0)	4 (17.4)	
Eye, n (%)				
Right	12 (60.0)	19 (46.3)	14 (60.9)	0.4303^b^
Left	8 (40.0)	22 (53.7)	9 (39.1)	
Type of patient, n (%)				
Naïve	6 (30.0)	13 (31.7)	10 (43.5)	0.5653^b^
Non-naïve	14 (70.0)	28 (68.3)	13 (56.5)	
Visual Acuity^**^				
Mean (SD)	64.8 (10.1)	58.8 (16.2)	60.9 (14.6)	0.3272
95% CI	60.0 to 69.5	53.7 to 63.9	54.5 to 67.2	
Number of anti-VEGF injections				
Mean (SD)	5.3 (4.9)	5.1 (4.9)	3.1 (3.2)	0.1877
95% CI	3.0 to 7.6	3.5 to 6.6	1.7 to 4.5	
CMT, μm				
Mean (SD)	352.6 (38.4)	459.4 (141.8)^*^	533.8 (149.2)^*^	< 0.0001
95% CI	334,6 to 370,6	414,7 to 504,2	469,3 to 598,3	

*DRT* diffuse retinal thickness, *CME* cystoid macular edema, *SRD* serous retinal detachment, *SD* standard deviation, *CI* confidence interval, *VEGF* vascular endothelial grow factor, *CMT* central macular thickness

^a^One-way ANOVA test

^b^Chi-square test

^*^Significantly different from DRT, *p* < 0.001

^**^Letters in the Early Treatment Diabetic Retinopathy Study (ETDRS) charts

**Fig. 1:**
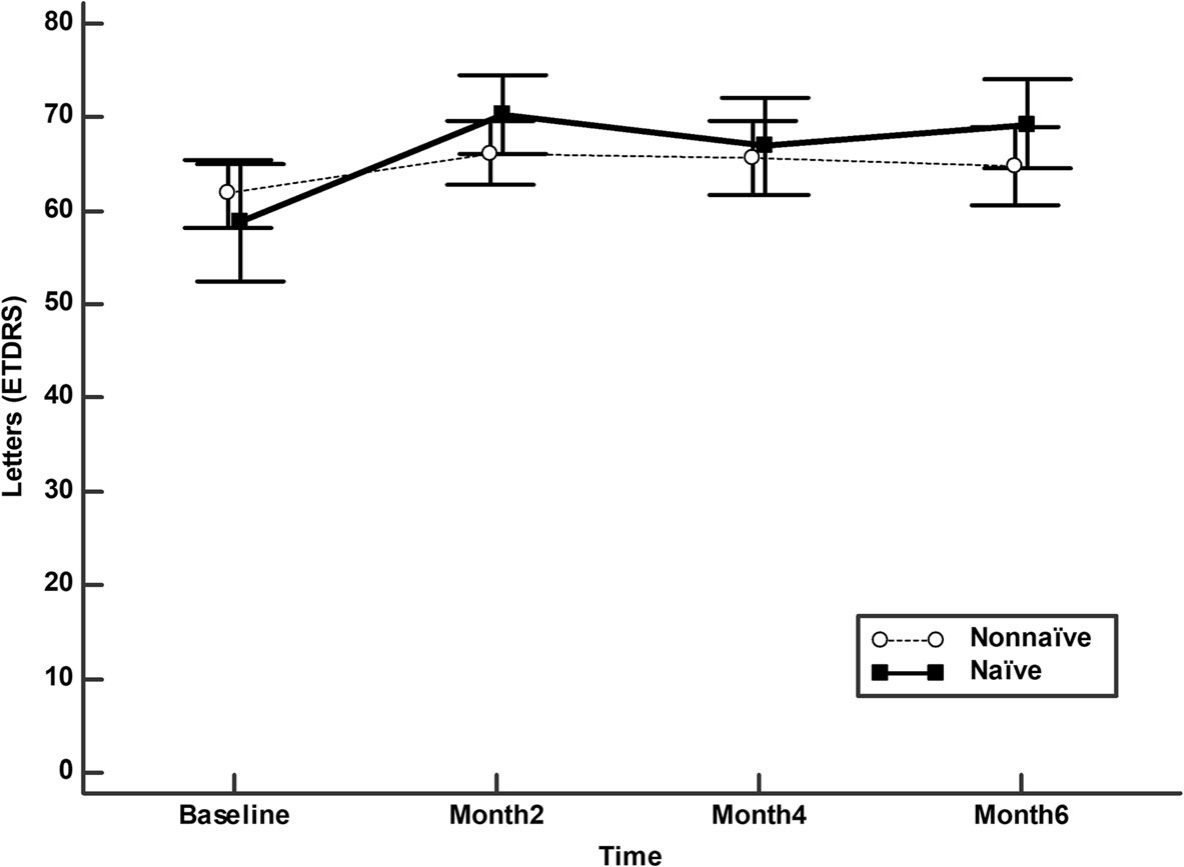
Mean best corrected visual acuity [Early Treatment Diabetic Retinopathy Study (ETDRS) charts] in naïve and Non-naïve patients. The vertical bars represent the 95% confidence interval

**Fig. 2:**
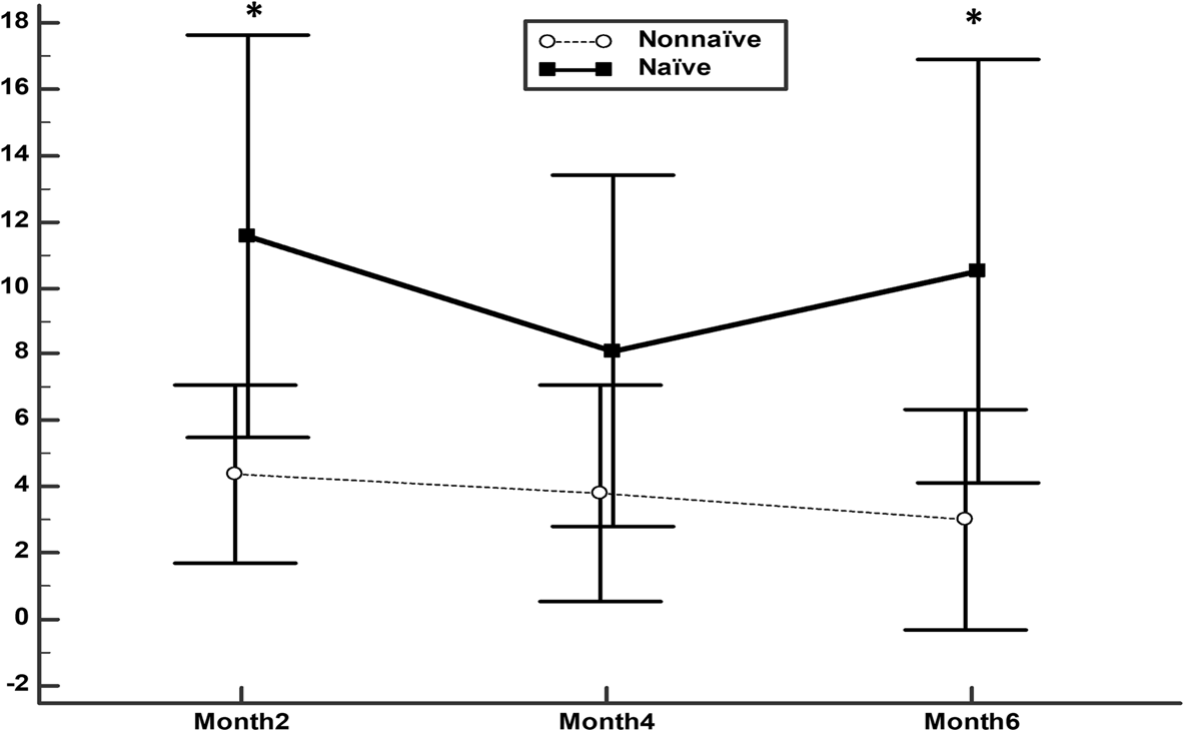
Mean change in best corrected visual acuity [Early Treatment Diabetic Retinopathy Study (ETDRS) charts] in naïve and Non-naïve patients. The vertical bars represent the 95% confidence interval. Statistical significance was determined using the Mann–Whitney U test (statistical significance *P < 0.05)

In the naïve patients the BCVA significantly improved from 58.8 (16.4) at baseline to 70.4 (11.2), 66.9 (13.6), and 69.3 (12.5) at 2,4, and 6 months of follow-up, respectively (*p* < 0.0001, Friedman rank sum test) (Fig. 1). In the non-naïve patients the BCVA significantly improved from 61.8 (13.6) at baseline to 66.2 (12.7), 65.6 (14.8), and 64.8 (15.5) at 2,4, and 6 months of follow-up, respectively (*p* = 0.0016, Friedman rank sum test) (Fig. 1). The improvement of BCVA at months 2 and 6 were significantly higher in the naïve patients as compared with the non-naïve ones (Fig. 2).

At the end of the study, the proportion of patients gaining ≥5 letters was 70.0 and 52.7% in the naïve and non-naïve patients respectively, *p* = 0.1281. Nevertheless, the proportion of patients gaining ≥10 letters was significantly greater in the naïve group than in the nonnaïve one (55.2 and 29.1%, respectively), *p* = 0.0199.

At baseline, the mean CMT was 466.2 (189.7) μm and 448.1 (110.7) μm in the naïve and non-naïve patients, respectively (*p* = 0.5830); and decreased to 339.3 (92.5) μm and 357.5 (79.1) μm, respectively (*p* = 0.0004 and *p* < 0.0001) Table 3.

**Table 3:** Mean changes in central macular thickness (CMT) in naïve and non-naïve patients. *P* values, between naïve and non-naïve patients, were calculated using the Mann–Whitney U test (statistical significance *P* < 0.05)

	Naïve	Non-Naïve	p
DifCMTM2, μm			
Mean	157.7	107.2	0.3714
95% CI	77.4 to 237.9	75.5 to 138.8	
DifCMTM4, μm			
Mean	132.4	68.8*	0.4158
95% CI	55.4 to 209.3	35.8 to 101.7	
DifCMTM6, μm			
Mean	126.9	90.7	0.2899
95% CI	62.0 to 191.8	56.6 to 124.7	
Intragroup Significance^**^	0.265	0.007	

*DifCMTM* difference in central macular thickness at month, *CI* confidence interval

^*^In the Non-naïve patients, the change in central macular thickness between months 2 and 4 was significantly different (Bonferroni corrected, *p* = 0.0016)

^**^In the naïve patients the change in macular thickness was stable and maintained over the course of follow-up. Whereas, in the non-naïve group there was a statistically significant difference between month 2 and 4. Repeated measures ANOVA and the Greenhouse-Geisser correction (Intragroup comparison)

The type of DME did not show any impact on the functional results. Nevertheless, the changes in CMT was significantly greater in the patients with SRD as compared with those with spongiform DME (Table 4). It might be due to the fact that the baseline CMT in the SRD patients was significantly thicker than in the DRT ones.

**Table 4:** Median change in best corrected visual acuity and central macular thickness according to the diabetic macular edema subtype diagnosed at baseline. P values were calculated using the Kruskal-Wallis test (statistical significance *P < 0.05)

	Median	25th percentile	75th percentile
	Changes in BCVA^1^		
DRT	2.5	0.0	10.0
CME	5.0	0.0	15.0
SRD	5.0	0.0	13.8
Significance	0.7479		
	Changes in CMT, μm		
DRT	34	- 5.5	107.0
CME	89	22.0	164.0
SRD	130*	59.8	216.5
Significance	0.0068		

*BCVA* best corrected visual acuity, *DME* diabetic macular edema, *DRT* diffuse retinal thickness, *CME* cystoid macular edema, *SRD* serous retinal detachment, *CMT* central macular thickness

^1^Letters in the Early Treatment Diabetic Retinopathy Study (ETDRS) charts

^*^Significantly different from DRT (*p* < 0.05)

Most patients in this study received only one injection (64.3%). During the follow-up, 30 (35.7%) eyes required an additional DEX implant, 14 (46.7%) were naïve eyes and 16 (53.3%) were previously treated eyes, *p* = 0,6122. Patients received a mean 1.4 (0.5) injections each, 1.3 (0.5) in the naïve eyes and 1.5 (0.5) in the previously treated ones, *p* = 0.0828.

The recurrence of DME was observed in 16 (55.2%) and 38 (69.1%) in the naïve and non-naïve patients, respectively. There were no significant differences in the number of patients experienced a recurrence in DME according to the baseline DME subtype [9 (45%); 13 (31.7%) and 8 (34.8) in the DRT, CME and SRD patients, respectively, *p* = 0.5926].

The Table 5 shows the type of recurrence according to the type of baseline DME in naïve and non-naïve patients. The type of recurrence did not differ according to the type DME diagnosed at baseline.

**Table 5:** Type of diabetic macular edema (DME) recurrence according to the type of DME diagnosed at baseline

	Type of recurrence				
	No Recurrence	DRT	CME	SRD	
	Naïve				
Type of DME					
DRT	4	2	0	0	6 (20,7%)
CME	5	3	5	0	13 (44,8%)
SRD	4	2	3	1	10 (34,5%)
	13(44,8%)	7(24,1%)	8(27,6%)	1(3,4%)	29
Significance^*^	0.0700				
	Non-Naïve				
Type of ME					
DRT	5	6	3	0	14 (25,5%)
CME	8	7	13	0	28 (50,9%)
SRD	4	0	2	7	13 (23,6%)
	17(30,9%)	13(23,6%)	18(32,7%)	7(12,7%)	55
Significance^*^	0.6267				

*DME* diabetic macular edema, *DRT* diffuse retinal thickness, *CME* Cystoid macular edema, *SRD* serous retinal detachment

^*^Chi-squared test

## Discussion

From the data at 6 months follow-up, we can see that the slow-release intravitreal dexamethasone implant, Ozurdex, shows efficacy for the treatment of DME, as both substantial improvements were registered in BCVA values, and significant reductions of CMT observed.

While comparing the previously treated eyes and the naive ones, we found that both groups had improvement in visual acuity and decrease in foveal thickness. Nevertheless, the improvement in BCVA at months 2 and 6 were significantly greater in the naïve patients. In general terms, the results of our study did not differ significantly of those previously published [18–29].

Regarding the improvement in BCVA, this study found significant differences between naïve and previously treated eyes at months 2 and 6. These findings agree with previously published studies that indicated a greater improvement in BCVA in naïve compared with non-naïve DME eyes [23, 31, 34, 35]. In the study published by Escobar-Barranco et al. [35], the visual acuity at baseline in naïve eyes was significantly better than in non-naïve ones. In our study, similarly to that published by Iglicki et al. [31], there was no significant difference at baseline visual acuity between the naïve and non-naïve eyes. This finding supports the hypothesis that naïve eyes benefit more from the applied treatment. Conversely, Chhablani et al. [36] found a similar improvement in visual acuity in treatment-naïve and previously treated patients.

In the overall patients 58.3 and 38.1% of patients demonstrated visual gains of more than 5 and 10 letters, respectively. These results are in line with those previously published [18–29, 31, 34–36].

Regarding the CMT, there was a significant difference at the last follow-up from baseline in naive eyes (*p* = 0.0004), as well as in previously treated eyes (*p* < 0.0001). This finding agrees with previous published studies [18–29, 31, 34–36].

The type of DME diagnosed at baseline did not have any influence in the type of DME recurrence.

This study found that the anatomical response in the eyes with SRD was significantly better than that observe in the spongiform DME, although the changes in BCVA were similar. A possible explanation might be the fact that the baseline CMT in the SRD patients was significantly thicker than in the DRT ones. On the other hand, this finding could be explained by the inflammatory nature of SRD previously described [37]. It was found a significant association between the intravitreal concentration of interleukin-6 and the presence of SRD [37]. However, other morphologic changes, such as retinal cystic changes or retinal swellings, were no significantly associated with the concentrations of intravitreal cytokines [37]. Conversely, Chhablani et al. [36] did not find any relationship between Ozurdex response and the type of edema on OCT.

To the best of our knowledge, there is not published papers evaluating the impact of the morphologic subtypes with OCT on the DEX implant outcomes. This study found that, although there were no significant differences in changes in BCVA between groups, SRD subtype was associated with a greater reduction in the CMT than the DRT subtype. These findings partially agreed with those reported by Koytak et al. [32] who found no significant differences between groups in BCVA, whereas the CME and SRD subtypes showed a greater reduction in CMT than the DRT subtype [32].

In contrast to trials that showed a required reinjection of Ozurdex® at 6 months [18], our results showed that only four eyes (4.8%) required additional treatment within 3 months and 26 (30.9%) eyes required additional treatment before 6 months. This finding is also in contrast with the results published by Zucchiatti et al. who reported that 88.8% of their patients required a second injection in month 6 [38]. Moreover, Unsal et al. [29] found that a second injection was recommended for all patients, although only four received the second injection within 6 months (other patients refused the second injection due to financial or other reasons). The results of our study are similar to those reported by Chhablani et al. [36] who observed that 72% of the patients did not require an additional implant. However, in contrast with their results, the percentage of eyes that required additional treatment during the follow-up period among naïve eyes was slightly greater in our study (46.7% vs 33.3%, respectively) [36].

This study has some limitations that should be noted; among them is its retrospective design. The second limitation is the single-center nature of the study, with a limited number of patients. Nevertheless, the sample size was calculated prior the study. Additionally, in this study group, there were significant differences between patients with naive and non-naïve eyes in terms of age and sex distribution. Finally, the follow-up period is relatively short and, hence, caution needs to be employed while deriving conclusions.

## Conclusions

This study suggests that the intravitreal dexamethasone implant (Ozurdex) is effective for the treatment of DME, even in refractory cases that have failed to respond to previous therapies. Moreover, the benefit was greater in eyes that were treatment naïve. In addition, this paper shows that patients with SRD treated with Ozurdex present significantly greater changes in CMT than spongiform DME. Further investigation is needed to elucidate the benefits of Ozurdex as first line treatment in this subtype of edema.

## Abbreviations

ANOVA: Analysis of variance
BCVA: Best corrected visual acuity
CME: Cystoid macular edema
CMT: Central macular thickness
DEX: Dexamethasone intravitreal implant
DME: Diabetic macular edema
DRT: Diffuse retinal thickness
ETDRS: Early Treatment Diabetic Retinopathy Study
FA: Fluorescein angiography
FDA: Food and Drug Administration
IRF: Intraretinal fluid
MV: Macular volume
OCT: Optical coherence tomography
PRN: Pro renata
SD: Standard deviation
SDm: Spectral domain
SRD: Serous retinal detachment
SRF: Subretinal fluid
VA: Visual acuity
VEGF: Vascular endothelial growth factor
